# Encapsulation-Dependent Enhanced Emission of Near-Infrared Nanoparticles Using *in vivo* Three-Photon Fluorescence Imaging

**DOI:** 10.3389/fbioe.2020.01029

**Published:** 2020-09-10

**Authors:** Ye Du, Nuernisha Alifu, Zhiyuan Wu, Runze Chen, Xiaozhen Wang, Guang Ji, Qian Li, Jun Qian, Bin Xu, Dong Song

**Affiliations:** ^1^Department of Breast Surgery, The First Hospital, Jilin University, Changchun, China; ^2^State Key Laboratory of Modern Optical Instrumentations, Centre for Optical and Electromagnetic Research, JORCEP (Sino-Swedish Joint Research Center of Photonics), Zhejiang University, Hangzhou, China; ^3^State Key Laboratory of Supramolecular Structure and Materials, Jilin University, Changchun, China

**Keywords:** AIEgen, organic dye nanoparticles, NIR fluorescence, three-photon fluorescence, *in vivo* brain imaging

## Abstract

We discovered a unique fluorescent enhancement of dye encapsulated polymeric nanoparticles, which strongly depended on the polymeric matrix. Interestingly, the polymer nanoparticles containing a NIR emissive dye exhibited remarkable enhancement of emission encapsulated by the polymer amphiphilic polymer containing polystyrene (PS) moiety, whereas the nanoparticles showed weak fluorescence when using other polymer encapsulation. The highest fluorescent quantum yield of nanoparticles can reach 27% by using PS-PEG encapsulation, where the strong NIR fluorescence can be observed. These ultra-bright fluorescence nanoparticles also possess a strong three-photon fluorescence and show a good candidate for *in vivo* vascular three-photon fluorescence imaging of mouse brain and ear under 1550 nm fs laser excitation. A fine three-dimensional (3D) reconstruction with an imaging depth of 635 and 180 μm was achieved, respectively. We further demonstrate that these nanoparticles can effectively target the sentinel lymph node (SLN) of mice.

## Introduction

Fluorescence imaging plays a very significant role in the fields of life science and biomedicine (Situ et al., 2016; Wang et al., 2017; Zhen et al., 2018)since it can offer high spatial resolution (Rust et al., 2006; Zhen et al., 2018) and sensitivity (Baddeley et al., 2011) to bio-samples. Deep tissue fluorescence imaging has attracted great research attention in clinical diagnosis and biomedical research, including functional brain imaging (Axelrod, 2001) sentinel lymph node mapping (Kobat et al., 2011; Qi et al., 2018; Zheng et al., 2018) and tumor targeting (Wu et al., 2017). The focusing ability and penetration depth of a light beam are limited by the optical absorption and scattering of biological tissue. Thus, the depth of fluorescence imaging is usually limited, which is a bottleneck in practical biomedical applications. Among these imaging techniques, near infrared (NIR) fluorescence imaging (Shao et al., 2015; Li et al., 2016; Song et al., 2016; Feng et al., 2017) and multi-photon excited fluorescence microscopy have become two deep-tissue fluorescence imaging approaches because of their potential abilities to overcome tissue absorption and scattering. Three-photon fluorescence microscopy, which has the advantages of a high signal-to-noise ratio, high penetration depth and spatial resolution, shines in the field of biological imaging.

Over the past few decades, various fluorescent materials, such as semiconductor quantum dots, fluorescent carbon dots, metallic nanoclusters and organic fluorescent small molecules and nanoparticles, have been developed and extensively investigated for biological imaging applications (Liu et al., 2019). Among them, fluorescent polymer nanoparticles have shown great potential in bioimaging, diagnostics, drug delivery and therapy owing to their unique optical and electronic properties, easy functionalization and excellent biocompatibility (Qi et al., 2016; Ma et al., 2017). However, conventional organic dyes suffer from several problems, such as a small Stokes shift and aggregation-caused quenching (ACQ) effects (Qian et al., 2009; Massin et al., 2011; Wang et al., 2011; Lu et al., 2016; Fateminia et al., 2017). AIE molecules, which were discovered by Tang et al. (Liu et al., 2011; Shi et al., 2012), are organic compounds with excellent emission properties in the aggregated state or solid state that provide a novel approach to achieve high emission by manipulating the aggregated state. Recently, many kinds of AIE-based nanoparticles have been developed by using different methods to form fluorescent nanoparticles, such as AIEgens encapsulated into a polymer matrix and covalently binding AIEgens to polymers (Wang et al., 2013, 2016; Zhang et al., 2015; Yan et al., 2016). However, until now, reports of fluorescent nanoparticles, such as those with aggregation-dependent and fascinating luminescent properties, have been rare, probably due to the difficulty of molecular design and aggregation structure manipulation. High performance and expanded applications are therefore anticipated. Guidelines are available for specific molecular design to fine-tune the luminescent characteristics using a specific aggregation model for developing efficient fluorescent nanoparticles that can be utilized in biological imaging (Alifu et al., 2017; Qian and Tang, 2017). In this paper, we present a successful example of high-performance NIR fluorescent nanoparticles via manipulation of an encapsulation polymeric matrix based on the AIEgen 2-(4-bromophenyl)-3-(4-(4-(diphenylamino)styryl)phenyl)fumaronitrile (TB). Owing to the excellent optical characteristics, with both a high fluorescence quantum yield reaching 27% and strong three-photon fluorescence, these nanoparticles showed great potential applications for *in vivo* three-photon imaging and sentinel lymph node (SLN) mapping. Following such a strategy, additional nanoparticles with high-infrared fluorescence emission can be reasonably designed, which will provide more dye selection for fluorescent imaging.

## Materials and Methods

TB was synthesized according to our previous work (Alifu et al., 2017). PS-PEG and other chemical regents which not specially mentioned were purchased from Sigma Inc., The deionized water (DI water, 18.2 MΩ cm resistivity) obtained from the system has been utilized in all the experiments Using a Shimadzu UV-3600 UV-vis spectrophotometer to measure UV-vis absorption spectra. Using a Malvern Zetasizer Nano ZS size analyzer to measure dynamic light scattering (DLS) and zeta potential at room temperature.

Fluorescence quantum yield was measured using a Hitachi F4500 spectrofluorophotometer. Rhodamine B (excitation wavelength: 365 nm) was used as a standard to determine the fluorescence quantum yields of the nanoparticles.

### Preparation of the Nanoparticles (NPs)

TB was dissolved in tetrahydrofuran (THF) to make a 1 mg/mL stock solution. Four polymers were dissolved in THF to form a 2 mg/mL polymer solution. First, 500 μL of polymer solution (PS-PEG, PSMA, PIMA, F127) and 100, 66.7, 50, 40, and 33.3 μL of the TB stock solution was placed into 2 mL Eppendorf tubes. Different amounts of THF were added to dilute to 1 mL uniformity with mixing and obtain mixtures with a weight ratio of TB to polymers of 1:10, 1:15, 1:20, 1:25 and 1:30. The mixture was rapidly added to 5 ml deionized water, and the nanoparticles were formed after ultrasonic treatment. The THF was evaporated by continuous blowing nitrogen on a 78°C hot plate. A 220 nm filter head was used to obtain a transparent red NP aqueous solution.

### Cell Culture

The cytotoxicity of TPABDFN-PSMA nanoparticles toward HeLa cells was evaluated by following the instructions of cell counting kit-8 (CCK-8). A total of 5000 cells/well in a 100 mL suspension were incubated in 96-well plates for 24 h. Then, 100 mL of fresh culture medium containing PS-PEG@TB NPs at various concentrations (ranging from 0 to 120 μg/mL) was added into each well. The cells were first incubated for 24 h, then remove the medium, and the cells were washed with PBS for 3 times. Finally, 100 mL medium containing CCK-8 (10%) was added to each well for 2 h, and using a microplate reader (Thermo, United States) to measure its absorbance at 450 nm.

### Measurement of the 3PL of PS-PEG@TB NPs

The three-photon fluorescence spectrum of PS-PEG@TB NPs measured by the optical system is shown in Figure 3A. Adopting a 1550 nm femtosecond laser (FLCPA-01C, Calmar Laser, 400 fs, 1 MHz) as the light source. The laser beam was focused on a cuvette containing the PS-PEG@TB NP solution via a lens (focal length: 5 cm). The 3PL was received vertically using an objective lens (25 × 1.05 NA) and then directed into the spectrometer (PG 2000, Ideaoptics Instruments) after filtering via a 980 nm short-pass filter.

### Optical System for 3PL Imaging

The 1550 nm fs laser was coupled to an upright confocal microscope (Olympus, BX61W1-FV1000). After passing through a scan lens and a tube lens, the laser beam was focused onto the sample by a water-immersed microscope objective (XLPLN25XWMP2, Olympus, 25 1.05 NA). The imaging sample could be a glass capillary tube filled with an aqueous dispersion of PS-PEG@TB NPs or the brain or ear of a live mouse. 3PL signals were epi-collected with the same objective and then passed through a customized 1035 nm short-pass dichroic mirror and a 590 nm long-pass filter (removing the excitation light and ambient noise). Then, the remaining fluorescence signals were collected using an external photomultiplier tube (HPM-100-50 Becker & Hickl GmbH) via non-descanned detection (NDD). Pictures were collected every 5 μm along the Z-axis, and 3D imaging was reconstructed by Z-scan stacks.

### *In vivo* Imaging of the Blood Vessels of the Mouse Brain

Microsurgery of the cranial window on the mouse brain was performed. Briefly, the mice were anaesthetized, and a small piece of skull was excised using a dental drill. Microsurgery was performed under sterile conditions to avoid infection and damage. The mice were then intravenously injected with 200 mL of a PBS (1×) solution of PS-PEG@TB NPs (1 mg/mL) and placed under the aforementioned multi-photon scanning microscope after being anaesthetized. To ensure that the mice could live well during the whole brain vascular imaging process, the body temperature of the mice was maintained at 37 ± 1°C during the experimental period. For the description of the immobilization of mouse heads and how the objective of the upright multi-photon scanning microscope was arranged to contact the mouse brain, we refer to Qian’s previous work (Qian et al., 2012). The 3PL signals of PS-PEG@TB NPs (from the brain blood vessels) were received using the 3PL imaging system.

### *In vivo* Imaging of the Blood Vessels of the Mouse Ear

Three hundred milliliters of PS-PEG@TB NPs in 1 × PBS solution (1 mg/mL) was intravenously injected into the mice. The mice were anesthetized and placed on a Petri dish with one ear attached to the coverslip and placed under the aforementioned optical system for 3PL imaging.

### SLN Mapping of Mice

To investigate the SLN mapping of PS-PEG@TB nanoparticles in mice, we intradermally injected 0.1 mL nanoparticles (in 5% glucose) into the left forepaw pad of nude mice who were then anaesthetized with pentobarbital at various times after the NP injection. The sedated animals were then imaged using the *in vivo* optical imaging system.

## Results and Discussion

### Preparation and Characterization of PS-PEG@TB NPs

**FIGURE 1:**
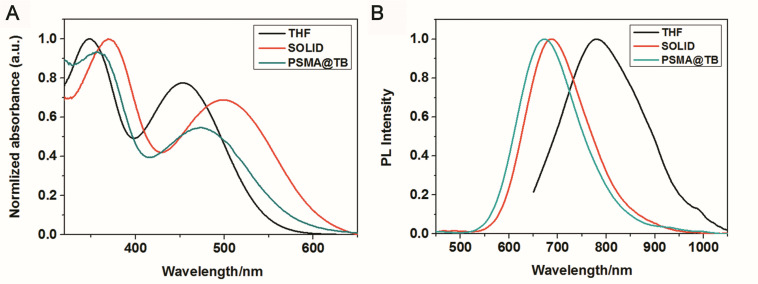
**(A)** UV–vis absorption and **(B)** PL emission spectra (excitation wavelength: 365 nm) of TB in THF (10–5 M) and in solid form and encapsulated with PSMA.

**SCHEME 1:**
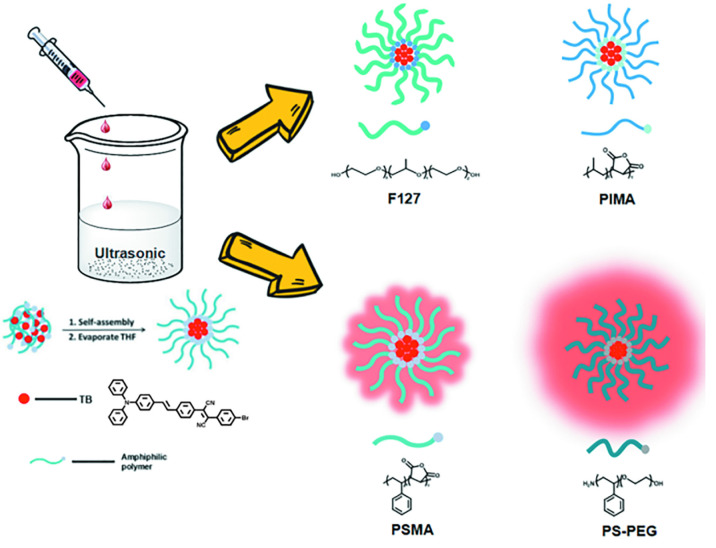
Schematic illustration of the preparation of TB loaded NPs.

**FIGURE 2:**
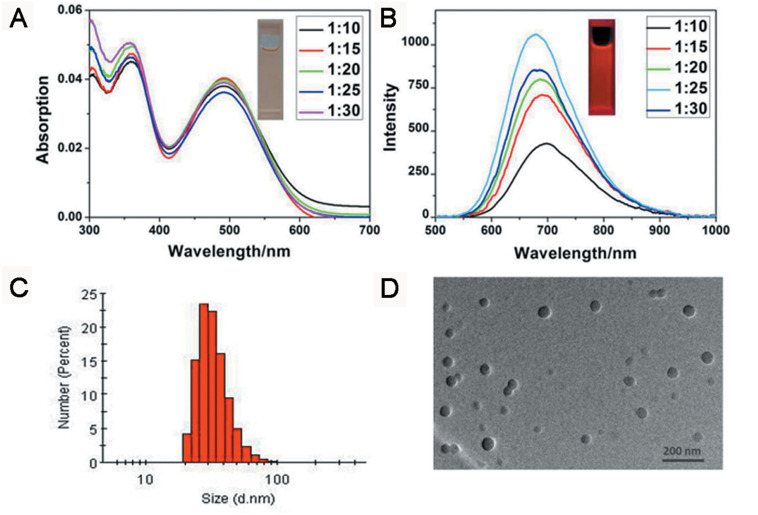
**(A)** UV-vis absorption and **(B)** PL emission spectra (excitation wavelength: 365 nm). **(C)** DLS distribution and **(D)** TEM image of PS-PEG@TB NPs.

TB could be synthesized by our previously reported method whose structure was fully verified by 1H NMR spectroscopy and high-resolution mass spectroscopy (HRMS) (Supplementary Figures S1, S2). As shown in Figure 1A, the TB has two main absorption peaks, mainly due to the π-π^∗^ transition (Figure 1A). The emission peak of TB reaches the NIR region, which is conducive to biological imaging (Figure 1B; Qian et al., 2012). By taking advantage of the amphiphilic properties of the copolymer, a water-dispersed polymeric micelle can be formed via self-assembly in aqueous solution (Scheme 1). Notably, the nanoparticles doped with TB showed weak emission in solution when encapsulated with the amphiphilic polymer F127. Moreover, the fluorescent quantum yield was approximately 1% when the concentration of dye was changed. This is quite different from most reported AIEgens, which exhibit strong emission after polymeric encapsulation (Ni et al., 2018). However, to our surprise, the nanoparticles showed strong emission when we used another amphiphilic copolymer, PSMA, containing a polystyrene moiety. The nanoparticles showed excellent optical properties, including a maximum absorption at 473 nm, the emission peaking at 671 nm, and more importantly, the fluorescent quantum yield can reach 20% by optimizing the ratio of dye to copolymer (Table 1). In addition, the luminescent feature of the nanoparticles encapsulated by PIMA was similar to those encapsulated by F127, which only changed the polystyrene moiety to polyisobutylene. As shown in Table 1, the fluorescent quantum yield of PIMA@TB nanoparticles showed a slight increase to 5%, which is still much lower than that of the PSMA@TB nanoparticles. These observations show that TB presents a unique luminescent feature when encapsulated by various amphiphilic copolymers. It is worth noting that the main difference between these copolymers is the benzene unit on the side chain of the polystyrene moiety, which shows more hydrophobic properties and larger steric hindrance than flexible alkyl and alkoxy chains. *When TB was mixed in a soft polymer matrix, the movement of the polymer segments at room temperature and relatively large free volume between the polymer chains enable the intramolecular motions of TB in the aggregate state. These intramolecular motions can consume excited-state energy and lack emission of TB.* Because TB is a multi-aromatic organic compound, the benzene unit may help the dye TB disperse well in the polymeric matrix due to moderate interactions between the polymer side chain and TB. *These intramolecular motions can be restricted when TB was dispersed in a rigid polymer matrix, allowing the molecules to decay via radiative channels and show strong emission. The proposed mechanism of AIE behavior is the restriction of intramolecular motions (RIM), which has been demonstrated in many typical AIE system.* Therefore, we suggest that TB shows an encapsulation-dependent enhanced emission feature, whose luminescent properties strongly depend on the side chain of the amphiphilic copolymer. In particular, the nanoparticles encapsulated by the polystyrene-containing copolymer boost efficient NIR emission, whereas the fluorescence of the nanoparticles was very weak with low efficiency. To demonstrate our hypothesis, we further fabricated nanoparticles encapsulated by the amphiphilic copolymer PS-PEG, which contains polystyrene and PEG segments. We optimized nanoparticles encapsulated with different polymers (Supplementary Figures S3–S6). Notably, the highest fluorescent quantum yield of 27% was reached when we optimized the doping concentration of the dye as well as the ratio of polymer to dye (Supplementary Figure S7). *The blue-shifted emission of nanoparticles encapsulated by PSMA and PS-PEG may originate from the enhanced intermolecular interactions between the benzene ring of polymer and TB, resulting the more twisted molecular conformation in the hydrophobic part.* As shown in Figure 2, the nanoparticles present some excellent optical properties, such as a maximum absorption at 483 nm and emission peaking at 665 nm. In addition, the τ of nanoparticles is 4.26 ns (Supplementary Figure S8) and the nanoparticles showed good dispensability with an average diameter of approximately 60 nm (Supplementary Figure S9) and an apparent zeta potential of -12.5 mV (Supplementary Figure S10), which are beneficial to biological imaging applications34 (Ni et al., 2018).

**TABLE 1:** Fluorescence quantum efficiency with different polymer proportions.

Different weight ratio (TB: copolymer)	Φ F127@TB	Φ PIMA@TB	Φ PSMA@TB	Φ PS-PEG@TB
1:10	1.3	5.0	13.9	8.2
1:15	1.2	5.6	14.7	17.7
1:20	1.1	4.5	18.1	20
1:25	1.3	4.4	20.0	27.1
1:30	1.3	4.2	19.6	21.5

### Cell Viability Analysis

Supplementary Figure S11 shows the relative viabilities of HeLa cells treated with PS-PEG@TB NPs after 24 h. The cells remained over 90% viable even when the concentration of the PS-PEG@TB NPs reached 120 μg/mL, indicating the low cytotoxicity of PS-PEG@TB NPs and further validating their application in bioimaging.

### *In vivo* 3PL Imaging of Mouse Ear Blood Vessels

**FIGURE 3:**
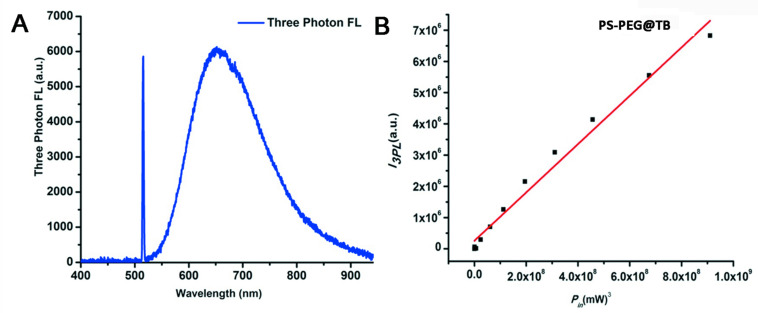
**(A)** Three-photon fluorescence spectra of PS-PEG@TB NPs under 1550 nm fs excitation. **(B)** Cubic dependence of the three-photon-induced fluorescence of PS-PEG@TB NPs on the excitation intensity of the 1550 nm-fs laser.

**FIGURE 4:**
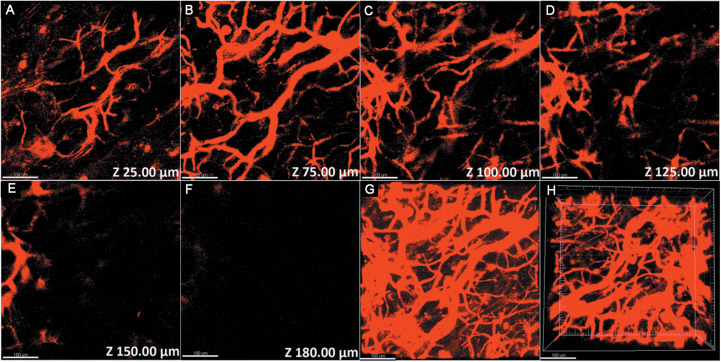
3PL imaging of ear blood vessels of a mouse treated with PS-PEG@TB NPs at various vertical depths: **(A)** 25 μm, **(B)** 75 μm, **(C)** 100 μm, **(D)** 125 μm, **(E)** 150 μm, and **(F)** 180 μm. **(G)** A stacked three-photon fluorescence image from a depth of 0 to 180 μm. **(H)** A 3D reconstructed image showing the distribution of the PS-PEG@TB NPs in the ear blood vessels of the mouse. Scale bar: 100 μm.

Owing to the excellent NIR luminescent properties of PS-PEG@TB, we explored their application in a home-built multi-photon system to record the non-linear optical response of the PS-PEG@TB NPs under the excitation of a 1550 nm fs laser. Bright 3PL was observed, accompanied by sharp third harmonic generation (THG), as shown in Figure 3A. The 3PL spectrum of PS-PEG@TB NPs was centered at 655 nm, giving a deep red emission. The inset in Figure 3A shows 3PL imaging of an aqueous dispersion of PS-PEG@TB NPs in a glass capillary tube under 1550 nm fs laser excitation. Red fluorescence can be clearly observed. The power dependence relationship of the PS-PEG@TB NPs was studied under 1550 nm fs excitation. As shown in Figure 3B, the fluorescence intensity of PS-PEG@TB NPs had a very good linear relationship to the cubic of the excitation intensity, indicating that 3PL would be the main non-linear optical process. First, we used a mouse ear blood vessel model to examine the capability of PS-PEG@TB NPs in *in vivo* 3PEFM imaging. Figures 4A–F shows the images of PS-PEG@TB NPs at various depths of the mouse ear skin under 1040 nm-fs excitation. Aside from the small capillaries located throughout the dermis, major veins and arteries located deeper within the dermis could also be observed. The overall distribution of blood vessels can be observed by stacking the images as shown in Figure 4G. Figure 4H shows a 3D reconstructed image of the blood vasculature network within a region of the ear dermis. *Otherwise, the fluorescent intensity of PS-PEG@TB nanoparticles under the 1550 nm laser irradiation show a little reduction over 90 min, demonstrating that PS-PEG@TB have excellent photostability as shown in* *Supplementary Figure S14*. These results indicated that PS-PEG@TB NPs hold great promise as an alternative contrast agent for intravital blood vasculature imaging.

### *In vivo* 3PL Imaging of Mouse Brain Blood Vessels

PS-PEG@TB NPs were further applied for the *in vivo* imaging of mouse brain blood vessels under the excitation of a 1550 nm fs laser. As shown in Supplementary Figure S13, PS-PEG@TB NPs emitted a bright 3PL signal in mouse brain blood vessels. As shown in Supplementary Figures S12A–G, the 3PL signal of PS-PEG@TB NPs clearly exhibit the structure of the mouse brain blood vessels at different depths, showing capillaries and even some of the fine structure. At imaging depths from 0 μm (Supplementary Figure S12A) to 385 μm (Supplementary Figure S12D), there are some mainly coarse vessels. At imaging depths from 485 μm (Supplementary Figure S12E) to 635 μm (Supplementary Figure S12G), the blood vessels observed were mainly capillaries. Supplementary Figure S12H is a stacked three-photon fluorescence image from a depth of 0 to 635 μm. A 3D mixed image of the PS-PEG@TB NPs in the blood vessels of the brain of a mouse was reconstructed as shown in Supplementary Figure S13. Reconstructing from different angles can restore the structure of mouse brain blood vessels to the greatest degree, which is beneficial for the exploration of brain science. The deep tissue imaging capability, high spatial resolution, high signal-to-noise ratio, and low thermal damage to biological samples make 3PL imaging very useful for brain vascular imaging of small animals.

### PS-PEG@TB NPs for SLN Mapping of Mice

**FIGURE 5:**
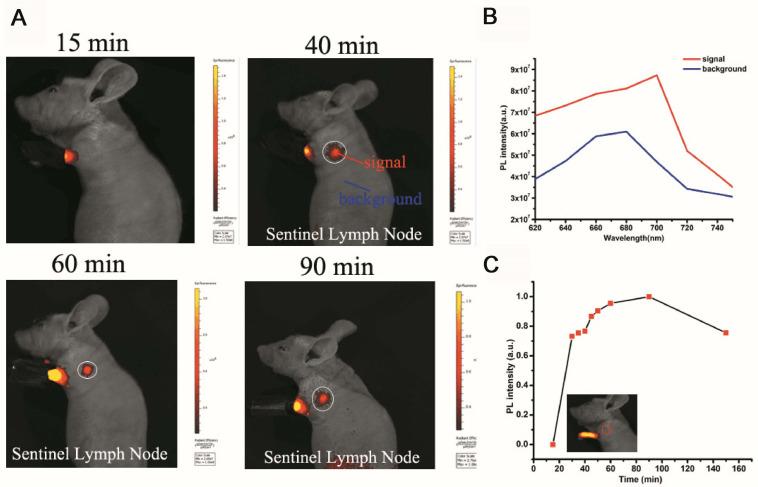
Sentinel lymph node (SLN) imaging of a nude mouse with PS-PEG@TB NPs intradermally injected into the forepaw pad at various times post-injection. **(A)** Fluorescence imaging (excitation light: 570 nm) of the mouse 15, 40, 60 and 90 min after injection. **(B)** The fluorescence intensity at different wavelengths with signal and background. **(C)** The fluorescence intensity of SLNs at different times post-injection.

The sentinel lymph node (SLN) can prevent lymphatic spreading out, so its clinical significance is very important. Figure 5A shows the SLN of nanoparticle imaging in mice. SLN is located in a white circle. The change in fluorescence intensity at the SLN after different injection times can be observed. Figure 5B shows a comparison of the luminescence signal at the SLN and the fluorescence signal of the other parts of the mouse (autologous background fluorescence). The results showed that the light intensity of the SLN signal is significantly higher than the background fluorescence signal, and it can be confirmed that the nanoparticles actually reached the SLN. Figure 5C shows the trend of the fluorescence intensity of the SLN after injection at different times. After 30 min, the signal slowly appeared in the SLN. As time went on, the nanoparticles continued to enter the SLN as the body fluid circulated, and the fluorescence intensity reached a maximum after 90 min. The fluorescence signal then began to decay. The signal becomes weak after 150 min. Through signal intensity analysis, this material can be used as a good lymph node contrast agent, which has potential clinical application value.

## Conclusion

The properties of these nanoparticles were strongly dependent on the composition of the encapsulated amphiphilic copolymer, where the polystyrene moiety can boost emission effectively compared with other non-aromatic polymeric segments. Hence, by using PS-PEG encapsulation to increase the ratio of the polystyrene moiety, the PS-PEG@TB NPs showed near-infrared emission at 665 nm with the highest fluorescent quantum yield of 27% and displayed excellent chemical stability, low toxicity and biocompatibility.

Furthermore, PS-PEG@TB NPs, which possess strong three-photon fluorescence, were utilized as fluorescent contrast agents for 3PL imaging under 1550 nm laser excitation, and for *in vivo* angiography of a mouse brain and ear, showing good imaging depths of 635 and 180 μm, respectively. We further demonstrated that these nanoparticles can effectively target the sentinel lymph node (SLN) of mice. This study virtually outlines an efficient encapsulation strategy for fabricating highly emissive organic nanoparticles and a high imaging depth multiphoton fluorescence imaging technique is applied *in vivo*. We believe that PS-PEG@TB NPs will become a good candidate for clinical deep-tissue bioimaging in the future.

## Data Availability Statement

All datasets presented in this study are included in the article/Supplementary Material.

## Ethics Statement

The animal experiments were performed strictly in compliance with the requirements and guidelines of the Institutional Ethical Committee of Animal Experimentation of Zhejiang University.

## Author Contributions

YD and BX: conception and design. JQ and GJ: administrative support. XW and QL: provision of study materials or patients. NA and ZW: collection and assembly of data. JQ and RC: data analysis and interpretation. DS: manuscript writing. All authors approved the final manuscript.

## Conflict of Interest

The authors declare that the research was conducted in the absence of any commercial or financial relationships that could be construed as a potential conflict of interest.
