# Supplementary material for: Encapsulation-Dependent Enhanced Emission of Near-Infrared Nanoparticles Using in vivo Three-Photon Fluorescence Imaging
Source: Front Bioeng Biotechnol. 2020 Sep 10;8:1029. doi: 10.3389/fbioe.2020.01029 (PMC7511574; doi:10.3389/fbioe.2020.01029)
Supplement: Supplementary file 1 [file Data_Sheet_1.pdf]

Supplementary Material

Figure S1. 1H NMR spectrum of TPABDFN.

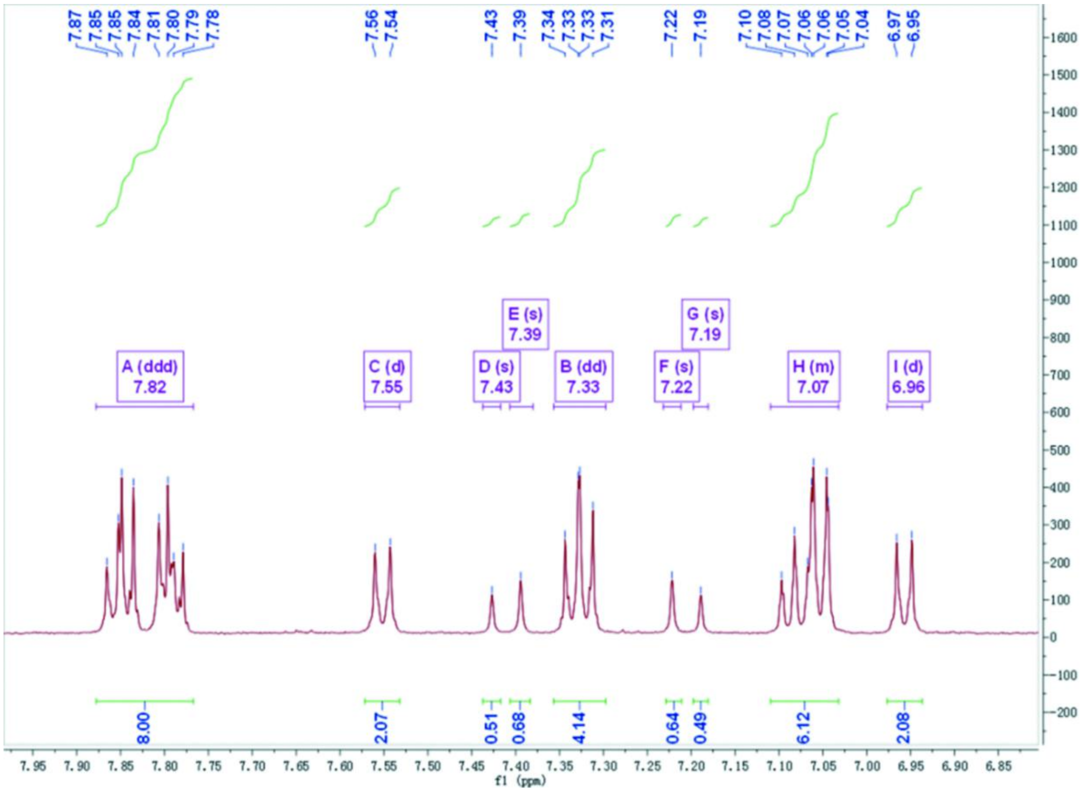

Figure S2. High-resolution mass spectrum (HRMS) of TPABDFN.

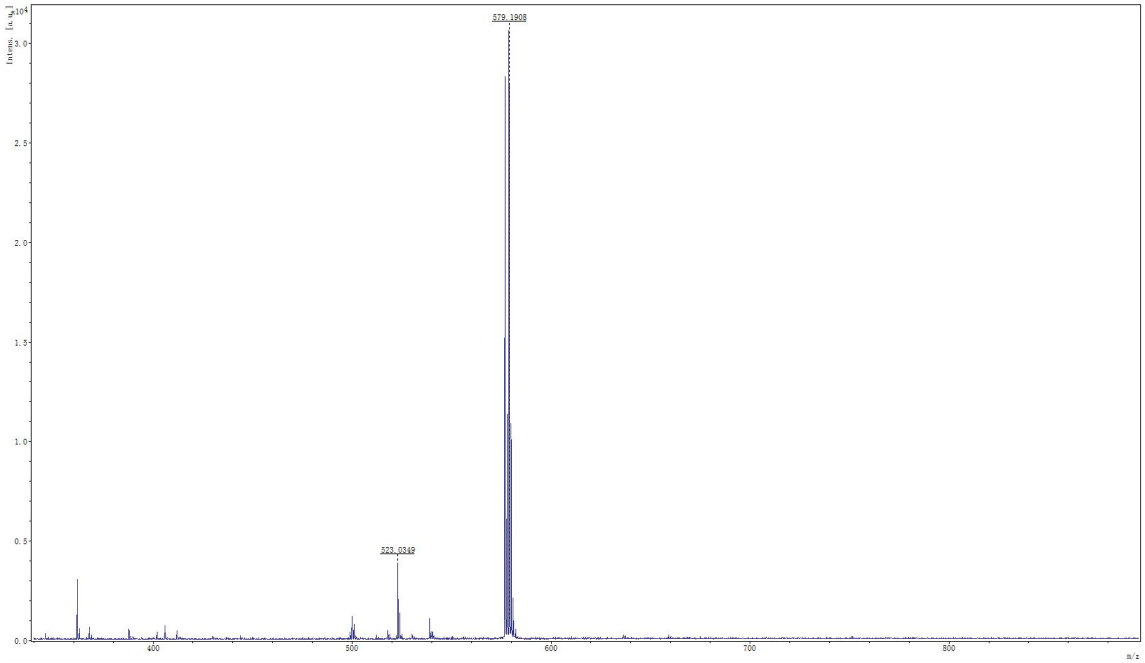

Figure S3. UV－vis absorption and PL emission spectra (excitation wavelength: 365 nm) of F127@TB NPs.

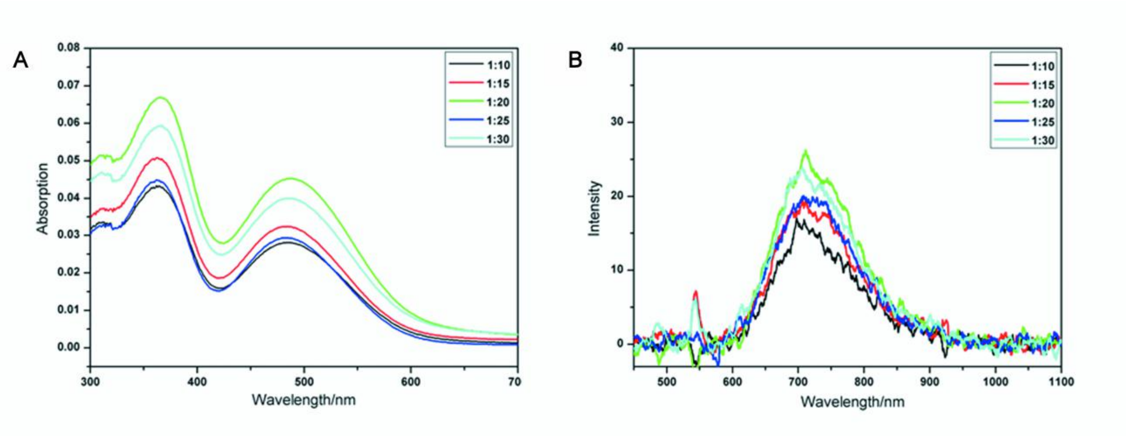

Figure S4. UV−vis absorption and PL emission spectra (excitation wavelength: 365 nm) of PSMA@TB

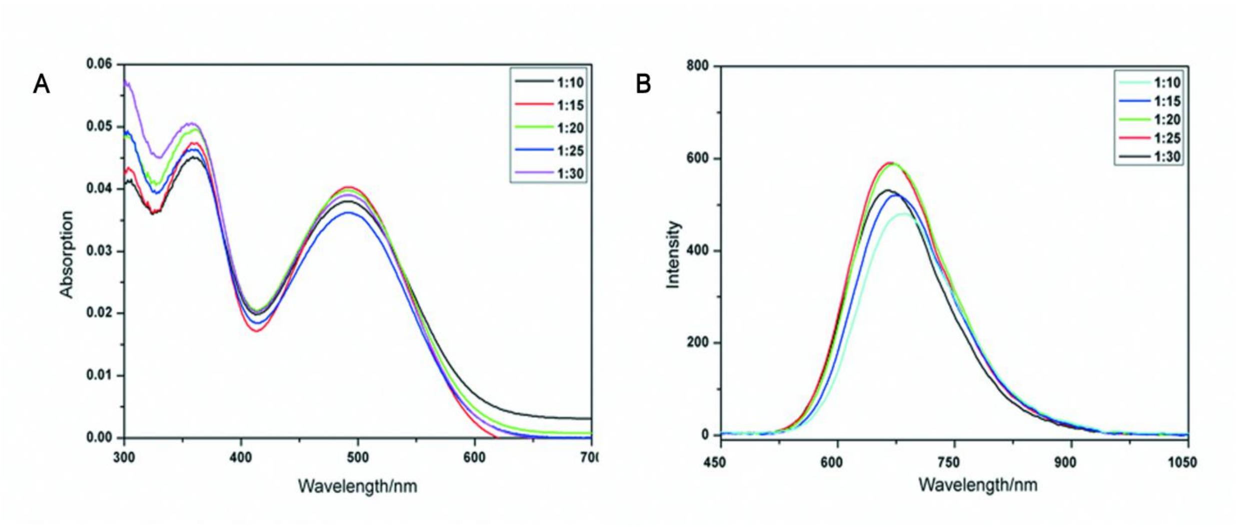

Figure S5. UV – vis absorption and PL emission spectra (excitation wavelength: 365 nm) of PSMA@2TBNPs

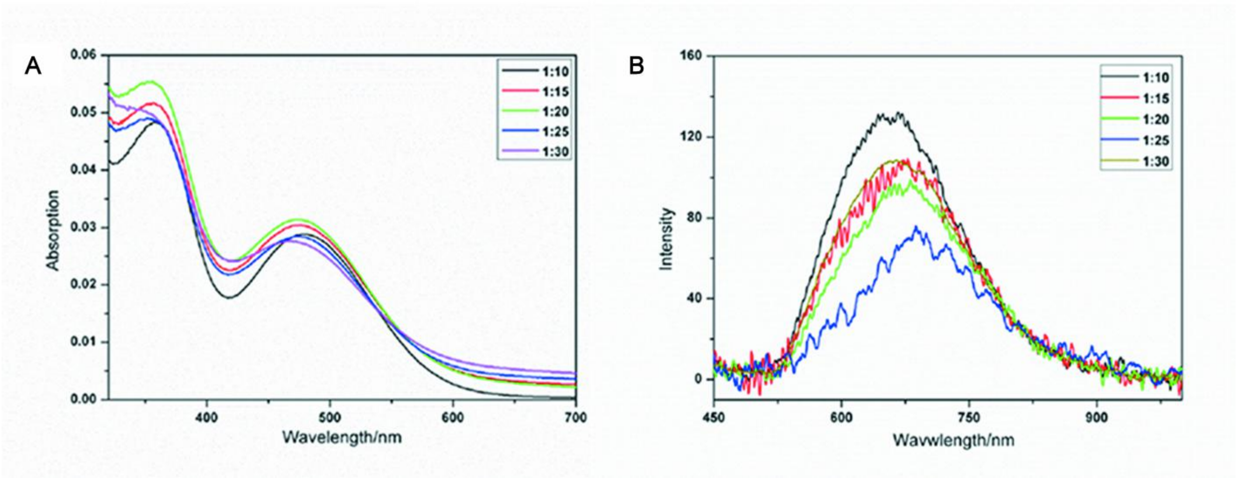

Figure S6. UV−vis absorption and PL emission spectra (excitation wavelength: 365 nm) of PS-PEG @TB

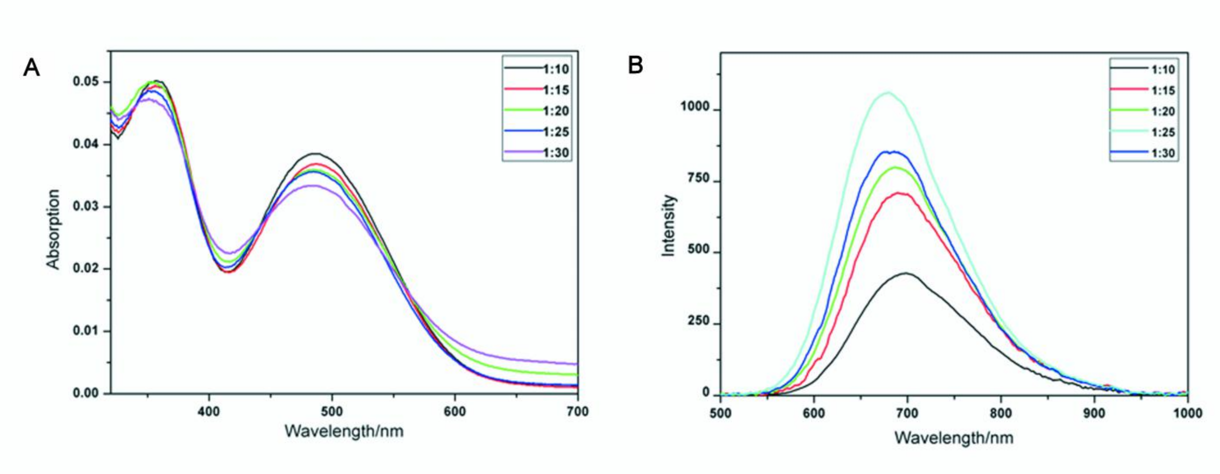

Figure S7. (A) Change trend chart of fluorescence quantum yield which different polymers encapsulated TB in a series of mass ratio. **(B)**Trend chart of maximum emission wavelength of different ratio polymers encapsulated dyes

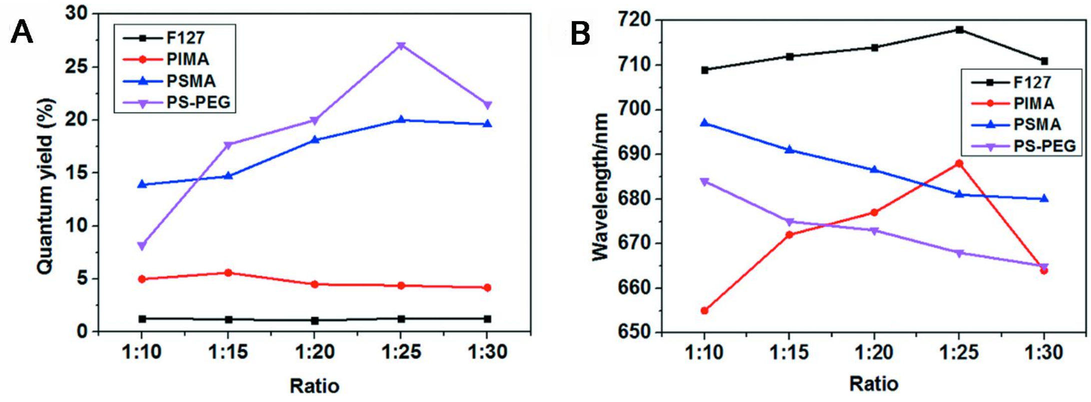

Figure S8. Time-resolved fluorescence spectra of different polymer encapsulated TB

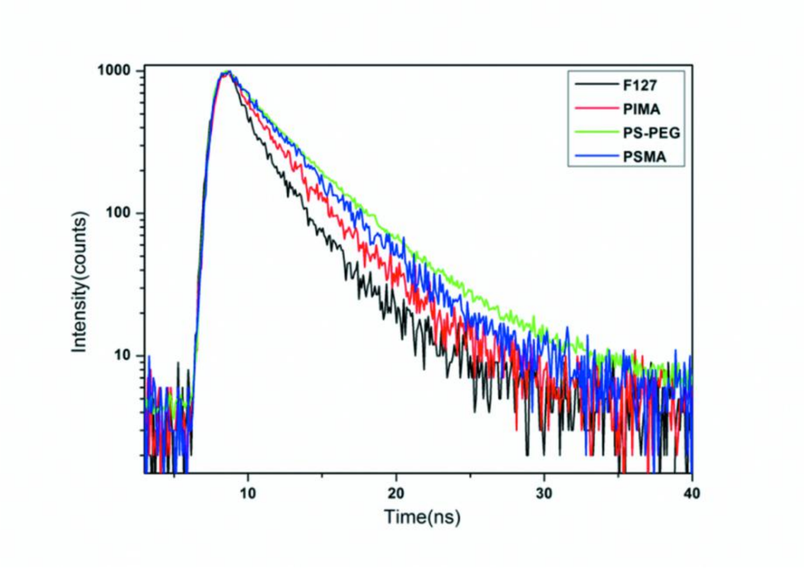

Figure S9. The DLS distribution of **(A)** PS-PEG@TB NPs, **(B)** PIMA@TB NPs, **(C)**F127@TB NPs and **(D)** PSMA@TN NPs. The average effective diameter are 61.0 nm, 67.5 nm, 50.3 nm and 66.1 nm respectively.

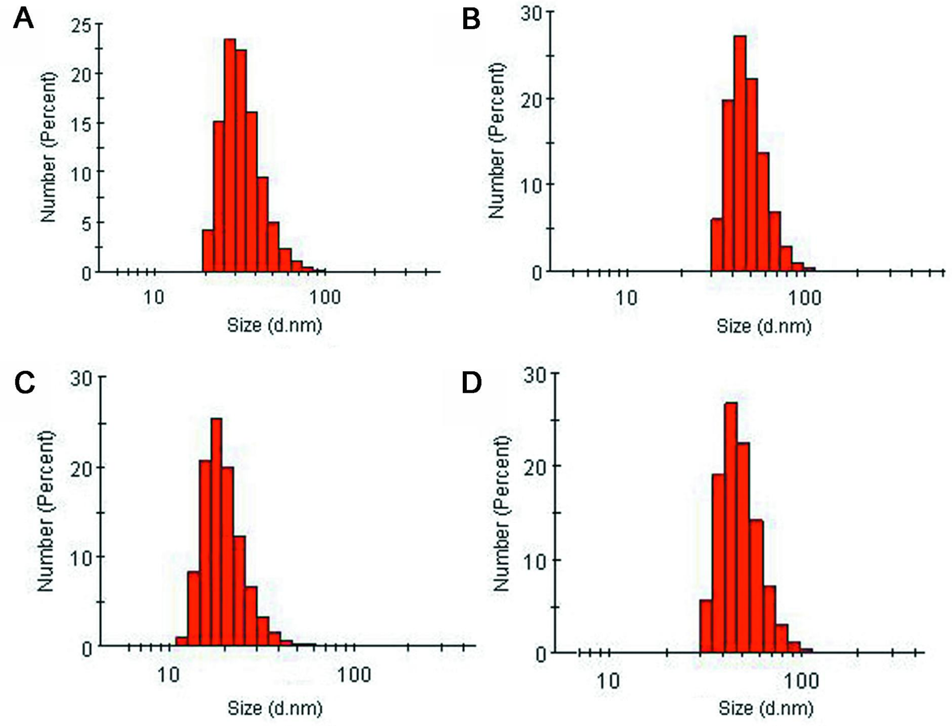

Figure S10. The Zeta potential distribution of **(A)** PS-PEG@TB NPs, **(B)**F127@TB NPs, **(C)**PSMA@TB NPs and **(D)**PIMA@TN NPs . The apparent Zeta potential are -12.5 mV, -11.6 mV, -44.6 mV and -35.7 mV respectively.

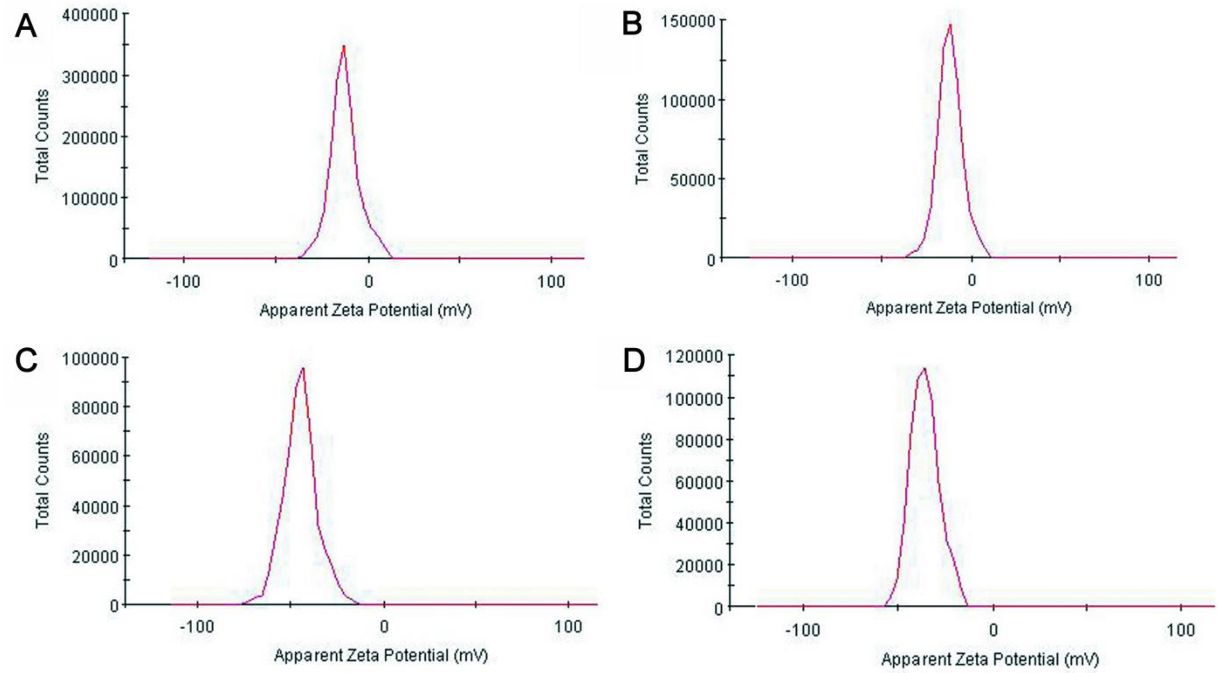

Figure S11. Viability of HeLa cells after incubation with PSMA@TB/NIR775 NPs with various concentrations for 24 h.

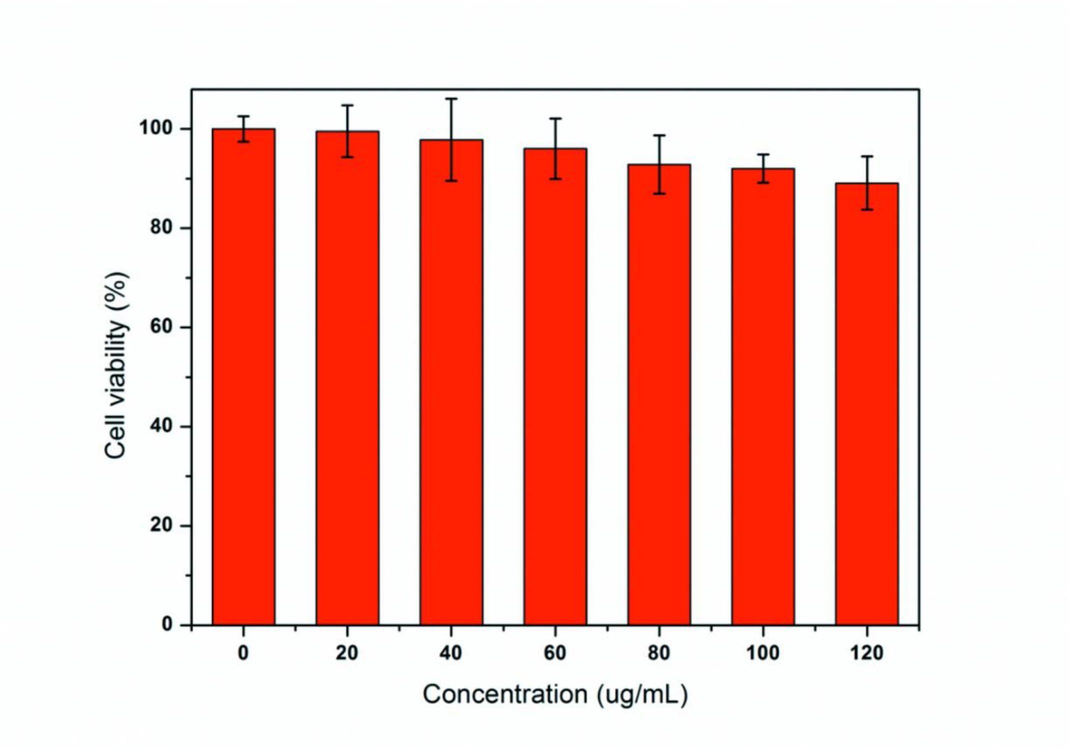

Figure S12. 3PL imaging of brain blood vessels of a mouse treated with PS-PEG@TB NPs, at various vertical depths: **(A)** 45 µm, **(B)** 195 µm, **(C)** 285 µm, **(D)** 385 µm, **(E)** 485 µm, **(F)** 585 µm and **(G)** 635 µm; **(H)** A stacked three-photon fluorescence image from a depth of 0 µm to 635 µm. Scale bar: 100 µm.

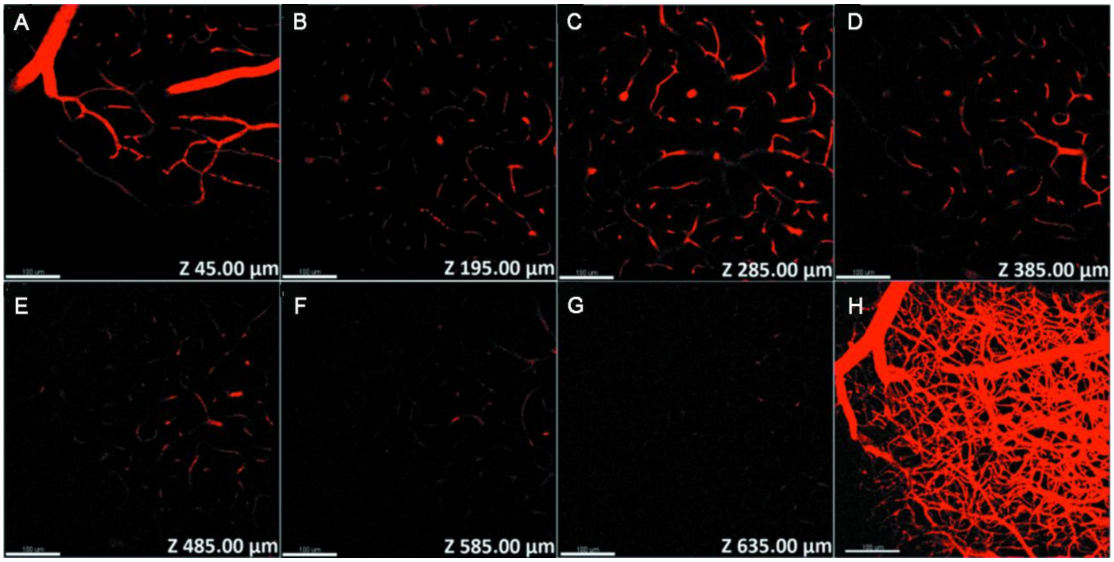

Figure S13. A 3D reconstructed image showing the distribution of the PS-PEG@TB NPs in brain blood vessels of the mouse (imaging depth: 635 μm). Scale bar: 100μm.

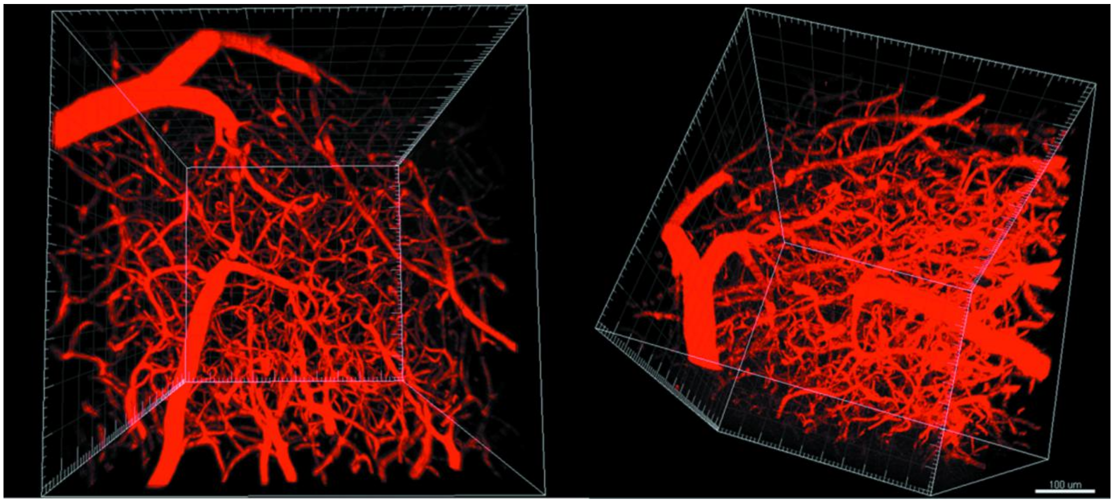

Figure S14. Photostability of nanoparticles encapsulated by PS-PEG upon two-photon laser irradiation. Excitation: 1550 nm.

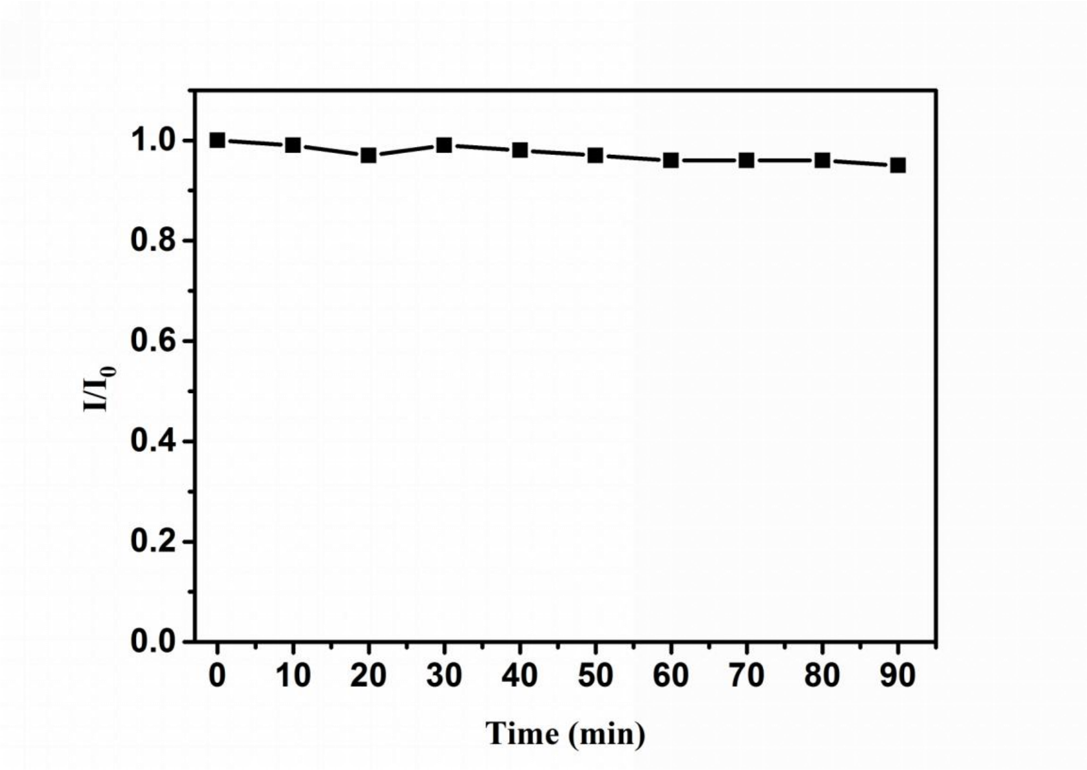

Table S1. The photophysical properties of nanoparticles.

	τ(ns)	k_r /10^7(s^{-1})	k_{nr} /10^8(s^{-1})
F127@TB	2.64	0.43	3.75
PIMA@TB	3.22	1.37	2.97
PSMA@TB	4.09	4.89	1.69
PS-PEG@TB	4.26	6.36	1.71
